# Glutamatergic Transmission: A Matter of Three

**DOI:** 10.1155/2015/787396

**Published:** 2015-08-04

**Authors:** Zila Martínez-Lozada, Arturo Ortega

**Affiliations:** Laboratorio de Neurotoxicología, Departamento de Toxicología, Centro de Investigación y de Estudios Avanzados del Instituto Politécnico Nacional, Apartado Postal 14-740, 07300 México, DF, Mexico

## Abstract

Glutamatergic transmission in the vertebrate brain requires the involvement of glia cells, in a continuous molecular dialogue. Glial glutamate receptors and transporters are key molecules that sense synaptic activity and by these means modify their physiology in the short and long term. Posttranslational modifications that regulate protein-protein interactions and modulate transmitter removal are triggered in glial cells by neuronal released glutamate. Moreover, glutamate signaling cascades in these cells are linked to transcriptional and translational control and are critically involved in the control of the *so-called* glutamate/glutamine shuttle and by these means in glutamatergic neurotransmission. In this contribution, we summarize our current understanding of the biochemical consequences of glutamate synaptic activity in their surrounding partners and dissect the molecular mechanisms that allow neurons to take control of glia physiology to ensure proper glutamate-mediated neuronal communication.

## 1. Introduction

Glutamate (Glu) the main excitatory neurotransmitter in the nervous system requires the involvement of neurons and glia cells to elicit its function as a neurotransmitter in an example of what is nowadays known as a* tripartite synapse*. Although detailed analysis of neuronal consequences of Glu exposure is regularly reviewed [1–3], the cellular and molecular impact of Glu in glia cells and its outcome in terms of synaptic communication are much less considered. Traditionally, it has been thought that Glu ejects its functions through the activation of specific membrane receptors classified in two main groups: ionotropic (iGluRs) and metabotropic (mGluRs). However, recent findings suggest the participation of Glu transporters in the signaling transactions triggered by this amino acid. Needless to say, both receptors and transporters are expressed in glia cells.

In the following sections, an insight into the signaling strategies used by this amino acid that end up into an efficient neuronal communication by means of altering the glial proteome is summarized and discussed.

## 2. Glutamate Receptors

Based on the sequence and transduction similarities, two main subtypes of Glu receptors have been defined: iGluRs and mGluRs. iGluRs are ligated-coupled ion channels that were originally classified according to their pharmacological profile into 5-methyl-4-isoxazole propionate (AMPA), kainate (KA), and N-methyl-D-aspartate (NMDA) receptors [4]. In terms of their molecular structure, each of these subtypes is composed of four subunits encoded by different genes. AMPA receptors are composed of different combinations of GRIA1 (GluA1), GRIA2 (GluA2), GRIA3 (GluA3), and GRIA4 (GluA4). Each combination displays different cation channel properties; for instance, the sole presence of one GluA2 subunit favours Na^+^ permeability. AMPA receptors lacking the GluA2 subunit are Ca^2+^-permeable [5], like those expressed in radial glia cells [6, 7]. KA receptors are composed of four subunits out of GluK1-5. While AMPA and KA receptors are homo- or heterooligomers NMDA receptors are formed as heteromers since in order to be functional they must contain at least one GluN1 subunit, with the other subunits of the channel being GluN2A-D or GluN3A-B subunits. The molecular diversity of these receptors is enormous, since most of these ionotropic subunits undergo RNA edition as well as splicing [8].

In contrast, mGluRs are members of class C of G-protein coupled receptors (GPCR) and had been classified based on sequence homology, G-protein coupled and pharmacology in three groups. Group I is comprised of mGluR 1 and mGluR 5 and is coupled to stimulation of phospholipase C with the consequent release of intracellular Ca^2+^, group II contains mGluR 2 and mGluR 3 and is coupled to adenylate cyclase inhibition, and group III consists of mGluR 4, mGluR 6, mGluR 7, and mGluR 8; as group II, group III is also linked to adenylate cyclase inhibition. These three groups are activated by specific agonist: for group I (RS)-3,5-dihydroxyphenylglycine (DHPG) and for group II (S)-4-carboxy-3-hydroxy-phenylglycine (S)-4C3HPG, while for group III L-(+)-2-amino-4-phosphonobutyric acid (L-AP4) [9].

## 3. Glial Glu Receptors

Glial cells express different types of Glu receptors depending on the brain region and the differentiation stage [10]. For example, AMPA receptors are expressed in astrocytes throughout the entire brain; however, their properties differ due to the differential GluA subunits expression. In cerebellum, retina, and brainstem, glial AMPA receptors lack GluA2 subunit; therefore, these channels are Ca^2+^-permeable (Table 1) [11–14]. AMPA-mediated Ca^2+^ influx has an important role in glial metabolism and structure. It has been demonstrated that AMPA receptors regulate the protein repertoire in Bergmann glial cells at transcriptional and translational levels, as will be discussed more broadly ahead [15–17]. Moreover, when Bergmann glia AMPA receptors are rendered Na^+^-permeable, their fusiform morphology changes through the retraction of the glial processes [18].

In contrast, although the transcripts and protein of various KA receptors subunits have been described in glial cells, no functional evidence of these receptors has been reported [19]. It has been speculated that KA receptors also participate in glial response to Glu since an increase of GluKs subunits expression is present in reactive astrocytes (Table 1) [20].

The expression of the seven known NMDA subunits has been demonstrated in glial cells (Table 1) [21–23], and glial NMDA receptors have peculiarities compared with neuronal NMDA receptors; glial NMDA receptors present a very weak Mg^2+^ blockage and a lower Ca^2+^ permeability [19, 24, 25].

In glial cells, mGluRs are also present; in fact astrocytes express all of the described subtypes, and mGluRs 1 and 5 from group I are linked to the activation of phospholipase C, while mGluRs 2 and 3 from group II and mGluRs 4, 6, 7, and 8 from group III are coupled to the inhibition of adenylate cyclase (Table 1) [26]. Calcium waves derived from stimulation of mGluR group I in glial cells have been shown and are considered as an important mechanism for Glu-dependent intracellular Ca^2+^ regulation in glial cells [27].

## 4. Membrane Glu Transporters

Glu extracellular levels are tightly regulated by a family of Na^+^-dependent Glu transporters known as excitatory amino acid transporters (EAATs) [78]. Five subtypes of Glu transporters have been characterized thus far and have been named EAATs 1–5. While EAAT 1 and EAAT 2 are regarded as glia specific, EAATs 3, 4, and 5 are present in neurons. The glial transporters EAAT 1, also known as Na^+^-dependent Glu/aspartate transporter (GLAST), and EAAT 2 (Glu transporter 1 (GLT-1)) are responsible of approximately 80–90% of Glu uptake activity in the brain [79], reflecting not only that glia cells outlast neurons in a 1 : 10 proportion, but also that these proteins are profusely expressed in glia cells. The neuronal transporters EAATs 3–5 have a more restricted distribution, EAAT 3 is expressed mainly in hippocampal neurons, and EAAT 4 is present in Purkinje cells in the cerebellum while EAAT 5 has been found in retina [78]. It should be noted, however, that EAAT 2 expression in neurons and EAAT 4 presence in astrocytes have also been documented [78, 80].

Glial Glu transporters are abundant; in fact it has been calculated that GLT-1/EAAT 2 represents 2% of total brain protein. While GLAST/EAAT 1 is preferentially expressed in cerebellum, retina, and olfactory bulb, GLT-1 is abundant in all other brain areas. During development, GLAST is the most abundant glial Glu transporter, and as such it has been widely used as a glial marker in numerous ontogeny studies (Table 2) [78].

These transporters have been traditionally implicated in Glu turnover through the* so-called* Glu/glutamine (Gln) shuttle. Once this amino acid is removed from the synaptic space, it is rapidly converted to Gln through the action of Gln synthetase [81, 82]. Sodium-dependent neutral amino acid transporters (SNATs) mediate both the glial release and the neuronal uptake of Gln, which once in the neuronal compartment is deaminated to regenerate Glu that is charged into the synaptic vesicles due to the action of the vesicular transporters (VGLUT).

A biochemical and physical coupling of GLAST with SNAT 3 was found in Bergmann glial cells, and we could demonstrate that the Na^+^ influx through GLAST activity is coupled to the Gln release mediated by SNAT 3 [83]; these results suggest that glial cells surrounding glutamatergic synapses sense neuron-derived Glu to promote a more efficient Glu recycling and in consequence an enhanced neuronal communication.

Recent evidences suggest that Glu transporters might also participate in the signaling transactions triggered by this amino acid. More than two decades ago, Amara and coworkers demonstrated a Glu-dependent Ca^2+^ influx via an unconventional mechanism that involved Glu transporters rather than Glu receptors in pituitary GH3 cells [84]; later on, different groups reported that Glu transporters translocation to the plasma membrane is regulated by the transporter itself [85, 86]. These findings added a novel regulatory mechanism for EAATs, recently expanded to include the membrane diffusion of the transporters which has been shown to modify the kinetics of excitatory postsynaptic currents [87].

In this context, Glu transporters have receptor-like properties; for example, in rat cortical astrocytes, L-Glu, D- and L-Aspartate, and transportable Glu uptake inhibitors increase p42/44^MAPK^ phosphorylation [88]. Glu transporters activity also impacts the PI3K/Akt/mTOR pathway, an important mechanism to regulate protein synthesis after glutamatergic stimulation [89, 90]. It also has been reported that Glu transporters have physical interaction with Na^+^/K^+^-ATPase and operate as a functional macromolecular complex to regulate glutamatergic neurotransmission [91–93].

## 5. Vesicular Glu Transporters

While the Glu release by glial cells has been well documented, the mechanisms involved in this release are still controversial. One of the proposed mechanisms is the activation of the reversal mode of EAATs [94]; the other one is the Glu vesicle-mediated, Ca^2+^-dependent release [95].

Three isoforms of vesicular Glu transporters (VGLUTs 1, 2, and 3) have been cloned and characterized in the brain; these isoforms have differential distribution and distinct roles, as expected [96, 97]. The expression of VGLUTs in neurons is well documented [97, 98]; however their presence in glial cells is still under discussion.

The first report of the presence of functional VGLUTs in glial cells was shown in 2004 (Table 2) [75]. Using rat visual cortex, cultured astrocytes these authors suggest the expression of VGLUTs based on the fact that pharmacological inhibition of VGLUTs reduces a Ca^2+^-dependent exocytosis Glu release from astrocytes. Moreover, VGLUT 3 overexpression results in an enhanced Ca^2+^-dependent Glu release [99]. It is important to mention that the biochemical machinery (for example, synaptobrevin II), needed for a vesicular Glu release has also been detected in cultured astrocytes [100]. A stimulus and Ca^2+^-dependent Glu release has been reported [101] favouring the idea of gliotransmitters regulated release. There are different subtypes of vesicles storing amino acids, peptides, and ATP in astrocytes. Despite these findings, a biochemical evidence of the VGLUTs expression in glial cells is still absent; Li and collaborators evaluated VGLUTs expression using Western blots and single-vesicle imaging by total internal reflection fluorescence microscopy concluding that their findings could not support an irrefutable evidence of VGLUTs expression in glial cells [102]. In this scenario, the molecular mechanisms involved in Ca^2+^-dependent Glu release are uncertain.

## 6. Glu-Dependent Gene Expression Regulation in Glia Cells

### 6.1. Transcriptional Control

The ability of glial cells to modify their transcriptional profile in response to Glu was one of the first questions asked after the expression of functional Glu receptors was fully characterized [103]. The increase in intracellular Ca^2+^ associated with Glu exposure in a plethora of glia cells preparations led to the search of the expression and DNA binding of several transcription factors such as Fos, Jun, and the cAMP response element-binding protein (CREB) [104–106]. The identification and characterization of downstream genes regulated by Glu in glia cells have started to emerge and systematic transcriptional studies have also been undertaken, for example, in Bergmann glia [107]. The overall picture is that the transcriptional pattern upon Glu stimulation varies from different glia subtypes, and in that sense the signaling cascade that regulates such effect is specific [108, 109].

### 6.2. Translational Control

Translation represents the final step in gene expression regulation. Translational control offers the advantage of rapid response to external stimulus to change gene expression profiles without the requirement of mRNA synthesis and transport. Protein synthesis is the most energy demanding process in cell physiology and given the fact that glia cells that surround glutamatergic synapses are engaged in Glu removal, a biochemical phenomenon that relies on the activity of the Na^+^/K^+^-ATPase, the idea that Glu could regulate protein synthesis has long been attractive. Indeed, Glu induces a biphasic effect in overall protein synthesis in cultured Bergmann glia [110]. Glu treatment modifies [^35^S]-methionine incorporation into newly synthesized polypeptides in a time dependent event marked by a decrease in [^35^S]-methionine incorporation 15 min after Glu exposure, but after 30 min this phenomenon starts to revert, returning to basal levels after 120 min. The ribosomal transit time (RTT), meaning the average time that a cell takes to synthetize a polypeptide [111], is augmented 7-fold in Glu-treated cultured Bergmann glia, event that is reverted after 120 min [112].

Translational control is mainly mediated by phosphorylation of several components of the translational machinery [113]. The initiation phase is a recurrent target of regulation, through the posttranslational modification of eukaryotic initiation factors (eIFs). The initiator methionyl-tRNA is conveyed to the ribosome assembly by eukaryotic initiation factor 2 (eIF2) complexed with GTP. The conversion of inactive eIF2-GDP to active eIF2-GTP by eIF2B is regulated by phosphorylation. eIF2 has three subunits (*α*, *β*, and *γ*). Glu exposure leads to serine 51 eIF2*α* phosphorylation, modification that converts eIF2 from a substrate to a competitive inhibitor of eIF2B [114]. This phosphorylation does not inhibit the general function of eIF2 but renders the protein defective in recycling, resulting in the inhibition of the initiation phase of protein synthesis.

Glu exposure also regulates translation elongation, again, decreasing protein synthesis by the inhibition of the ribosomal translocation. It should be noted that regulation of elongation process is indicative of a transient effect since the mRNA remains attached to the ribosomes allowing an immediate reinitiation of the translation process, as shown in Bergmann glia [110].

In summary, a cascade of phosphorylation/dephosphorylation of translation factors is involved in the Glu biphasic translational control in cultured Bergmann glia. Exposure to this excitatory amino acid reduces in the first 15 min [^35^S]-methionine incorporation into trichloroacetic acid- (TCA-) precipitable polypeptides. Thereafter, a gradual recovery in protein synthesis starts, and after 120 min, the translation process has returned to control levels, suggesting that Glu regulates both translation initiation and elongation. Indeed, phosphorylation of the eIF2*α* is present after 10 min Glu exposure [115]. Glu also regulates the phosphorylation of eukaryotic elongation factor 2 (eEF2) via AMPA and KA receptors at this time frame [15]. Both phosphorylation events (eIF2*α* and eEF2) are time dependent events, with a kinetics that matches the downregulation of the protein synthesis upon Glu.

The recovery phase of protein synthesis in Glu exposed cells involves an increase in the mechanistic target of rapamycin (mTOR) phosphorylation [17, 90]; this protein is thought to act as a check point that regulates cellular translational capacity since this kinase is capable of transducing extracellular growth factors signals and by these means regulating translation. Once activated (phosphorylated) this kinase favours eukaryotic elongation factor 1A (eEF1A) phosphorylation, needed for translation reinitiation. After 60 min of Glu exposure, an increase of eukaryotic elongation factor 2 kinase (eEF2K) phosphorylation is present [15]. eEF2K phosphorylation is carried out by p90^RSK^ and by p70^S6K^ inhibiting its activity and therefore reducing eEF2 phosphorylation levels favouring ribosomal translocation and protein synthesis reinitiation.

### 6.3. Physiological Consequences of Glutamate-Dependent Protein Synthesis Regulation

Glu biphasic effect in protein synthesis is clear; but what is the physiological importance of this regulation? It is tempting to speculate the downregulation of protein synthesis as a consequence of a massive Glu exposure of glia cells surrounding glutamatergic synapses with the expected metabolic stress of the removal of the neurotransmitter from the synaptic cleft. It is important to mention that Glu concentration can reach a 0.1 mM concentration, well above of the *K*_*M*_ of the glial transporters, which is around 30 *μ*M [28]. Therefore a strict coupling with the Na^+^/K^+^-ATPase is present [92, 93]. It is clear then that under periods of sustained synaptic activity glial cells reduce their protein synthesis, in order to restore the Na^+^ gradient compulsory for neurotransmitter uptake. Besides the Glu uptake, its recycling also consumes energy. Neuronal Glu pools are replenished through the Glu/Gln shuttle [116].

The reduction of the overall elongation process is frequently linked to the translation of mRNAs with complex structures in their 3′ and 5′ untranslated regions (UTRs). Since translation initiation factors are accumulated and can interact with complex UTRs, in this scenario, Glu also has an important role in the regulation of the translation of specific mRNAs. One of the targets of this type of regulation is the Gln synthetase mRNA, and the interruption of the elongation process favours the translation of Gln synthetized mRNA needed for the referred shuttle [117].

## 7. Astrocyte-Neuron Lactate Shuttle

More than twenty years ago new evidences of another role of glial cells emerged, the astrocyte-neuron lactate shuttle (ANLS) [118]. As has been mentioned before, Glu uptake increases Na^+^ intracellular concentrations leading to the activation of Na^+^/K^+^-ATPase forming part of a macromolecular complex. The consumption of ATP activates glycolysis, with the consequent glucose utilization and lactate production. Lactate is released through the action of monocarboxylate transporters (MCT) 1 and 4 that are present in glial cells; once released, lactate is taken up by neurons through MCT 2 and used as an energy substrate. In this scenario glial cells have mainly glycolytic metabolism while neurons display an oxidative metabolism [119].

Although at the beginning this model raised controversies, genomic and metabolic approaches have shed some light on its importance and it is now well accepted. There are enough evidences of a metabolic compartmentalization between glial cells and neurons; first, Glu treatment in glial cells enhances glucose transport [118] with an increase in glucose consumption [120–122]. Enzyme lactate dehydrogenase (LDH) isoform 5 that favours lactate production is preferentially expressed in astrocytes, as well as the lactate transporters MCT 1 and 4 that have low affinity to lactate [123, 124], and astrocytes and endothelial cells express glucose transporter 1 (GLUT1). On the other hand, Glu decreases glucose transport in neurons [125], neurons express preferentially isoform 1 of the LDH that favours the lactate conversion to pyruvate and MCT 2, a transporter with high affinity to lactate [126], and express glucose transporter 3 (GLUT3) [127, 128].

## 8. Clinical Implications

It has been documented that loss of glial cells-dependent Glu homeostasis is a prerequisite for excitotoxicity. Glu release from astrocytes has clear pathophysiological implications, ranging from ischemic lesion such as stroke, to white matter injury through demyelinating disorders like multiple sclerosis, to dementias such as Alzheimer's and Huntington diseases [129]. Although the causative role of aberrant glutamate uptake in these diseases is not always supported by published data, downregulation of GLAST and GLT-1 expression has been correlated with cognitive deficits associated with the diseases mentioned before [130]. Decrease expression and function of GLAST and GLT-1 also correlates with cognitive deficits observed in heavy metal exposure, as lead (Pb) and methylmercury [131]. The reduced GLT-1 expression seen in ALS, schizophrenia, mood and anxiety disorders, Alzheimer's disease, brain injury, glaucoma, HIV-associated dementia, and addition is regulated at two levels, transcriptional and during mRNA maturation (splicing) [132]; for example, in ALS an abnormal splicing of Glu transporters mRNA which results in truncated mRNA species has been demonstrated [133]. Furthermore, aberrant Glu transporters expression and function are common to gliomas in order to favour their own growth, invasion, and survival [134], favouring the notion of protein repertoire regulation in glial cells.

## 9. Conclusion

**Figure 1:**
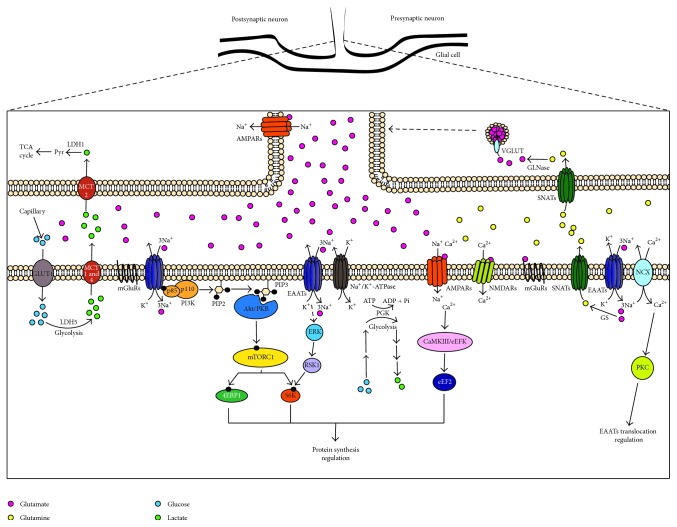
*Model of glutamatergic synapses with the role of the three components, presynaptic neuron, postsynaptic neuron, and glial cell*. Through the activation of glutamatergic receptors and transporters expressed in glial cells, these cells sense synaptic activity and regulate their protein repertoire through transcriptional and translational regulation, as well as translocation to plasmatic membrane. Some of the main contributions of glial cells to glutamatergic neurotransmission include the astrocyte-neuron lactate shuttle and Glu recycling through the Glu/Gln cycle.

Glial cells sense glutamatergic synaptic activity through Glu receptors and transporters and change their protein repertoire regulating transcription as well as translation of proteins critically involved in their continuous molecular dialogue with neurons. The Glu/Gln and the astrocyte-neuron lactate shuttles are the biochemical signature of coupling and are summarized in Figure 1. Much is left to be learned about the fine regulation of glutamatergic neurotransmission but one thing is for sure that glial cells have an active participation in this process.

## Figures and Tables

**Table 1:** Glial glutamate receptors. Summary of described glial glutamate receptors.

Glu receptor	Preparation	Reference
AMPA/KA without GluA2	BGC	[7, 11, 13]
AMPA	Oligodendrocytes precursor cells (OPCs)	[28, 29]
AMPA/KA	Cortical astrocytes	[30, 31]
AMPA/KA	Oligodendrocytes	[32]
GluA1, A2, A3, A4	Astrocytes isolated from CA1 region	[33]
GluA4, GluK5	Perivascular astrocytic processes	[34]
GluA2, A3, A4, A5, GluK1, K2	Microglia	[35]
KA Ca^2+^-permeable receptors	Glial cells of mouse hippocampal slices	[36]
KA Ca^2+^-permeable receptors	BGC	[7]
GluK1, K2	Astrocytes and oligodendrocytes	[37]
GluN1	Müller glial cells	[38]
GluN1, N2A, N2B	Cortical astrocytes	[39–41]
GluN1, N2A, N2B	BGC	[42]
GluN1, N2A, N2B, N2C, N2D, N3A, N3B	Human primary astrocytes	[43]
NMDA	OPCs, immature and mature oligodendrocytes in the white matter of cerebellum and corpus callosum	[44, 45]
AMPA, KA, NMDA	Glial cells in rat spinal cord slice	[46]
mGluR 5	Astrocytes	[47, 48]
mGluR 2, mGluR 3	Glial cells in several regions of the brain	[49, 50]
mGluR groups I and II	Human astrocytes	[51]
mGluR groups I, II, and III	OPCs and oligodendrocytes	[52]
mGluR	Microglia	[47, 53, 54]

**Table 2:** Glutamate transporters in glial cells. Summary of glutamate transporters described thus far.

Glu transporter	Preparation	Reference
GLAST/EAAT 1	Human cerebellar mRNA	[55, 56]
GLAST/EAAT 1	BGC	[57–59]
GLAST/EAAT 1	Müller glial cells	[60–63]
GLAST/EAAT 1	Activated microglia	[64]
GLT-1a and GLT-1b/EAAT 2	Astrocytes	[57, 65–67]
GLAST/EAAT 1, GLT-1/EAAT 2	Oligodendrocytes	[68–71]
GLT-1/EAAT 2	Microglia	[72]
EAAC1/EAAT 3	Astrocytes of the cerebral cortex	[73]
EAAC1/EAAT 3	OPCs	[69]
EAAC1/EAAT 3	Oligodendrocytes	[70]
EAAC1/EAAT 3	NG2^+^ cells	[74]
VGLUT 1	Astrocytes in culture	[75, 76]
VGLUT 2	Astrocytes in culture	[75]
VGLUT 2 and VGLUT 3	Astrocytes of the cortex and caudate-putamen	[77]
